# A Comparative Evaluation of Unsupervised Anomaly Detection Algorithms for Multivariate Data

**DOI:** 10.1371/journal.pone.0152173

**Published:** 2016-04-19

**Authors:** Markus Goldstein, Seiichi Uchida

**Affiliations:** 1 Center for Co-Evolutional Social System Innovation, Kyushu University, Fukuoka, Japan; 2 Department of Advanced Information Technology, Kyushu University, Fukuoka, Japan; Wayne State University, UNITED STATES

## Abstract

Anomaly detection is the process of identifying unexpected items or events in datasets, which differ from the norm. In contrast to standard classification tasks, anomaly detection is often applied on unlabeled data, taking only the internal structure of the dataset into account. This challenge is known as unsupervised anomaly detection and is addressed in many practical applications, for example in network intrusion detection, fraud detection as well as in the life science and medical domain. Dozens of algorithms have been proposed in this area, but unfortunately the research community still lacks a comparative universal evaluation as well as common publicly available datasets. These shortcomings are addressed in this study, where 19 different unsupervised anomaly detection algorithms are evaluated on 10 different datasets from multiple application domains. By publishing the source code and the datasets, this paper aims to be a new well-funded basis for unsupervised anomaly detection research. Additionally, this evaluation reveals the strengths and weaknesses of the different approaches for the first time. Besides the anomaly detection performance, computational effort, the impact of parameter settings as well as the global/local anomaly detection behavior is outlined. As a conclusion, we give an advise on algorithm selection for typical real-world tasks.

## Introduction

In machine learning, the detection of “not-normal” instances within datasets has always been of great interest. This process is commonly known as anomaly detection or outlier detection. The probably first definition was given by Grubbs in 1969 [1]: “An outlying observation, or *outlier*, is one that appears to deviate markedly from other members of the sample in which it occurs”. Although this definition is still valid today, the motivation for detecting these outliers is very different now. Back then, the main reason for the detection was to remove the outliers afterwards from the training data since pattern recognition algorithms were quite sensitive to outliers in the data. This procedure is also called *data cleansing*. After the development of more robust classifiers, the interest in anomaly detection decreased a lot. However, there was a turning point around the year 2000, when researchers started to get more interested in the anomalies itself, since they are often associated with particular interesting events or suspicious data records. Since then, many new algorithms have been developed which are evaluated in this paper. In this context, the definition of Grubbs was also extended such that today anomalies are known to have two important characteristics:

1. Anomalies are different from the norm with respect to their features and
2. They are rare in a dataset compared to normal instances.

Anomaly detection algorithms are now used in many application domains and often enhance traditional rule-based detection systems.

**Intrusion detection** is probably the most well-known application of anomaly detection [2, 3]. In this application scenario, network traffic and server applications are monitored. Potential intrusion attempts and exploits should then be identified using anomaly detection algorithms. Besides this network-based intrusion detection, also host-based intrusion detection systems are available, commonly using system call data of a running computers. Most security vendors often call anomaly detection in this context *behavioral analysis* [4]. An important challenge in these often commercial Intrusion Detection Systems (IDS) is the huge amount of data to be processed in near real-time. For this reason, these systems typically use simple but fast anomaly detection algorithms. Intrusion detection systems are also a good example where anomaly detection complements traditional rule-based systems: They typically use pattern matching for the fast and reliable detection of known threats while an additional anomaly detection module tries to identify yet unknown suspicious activity.

**Fraud detection** is another application of anomaly detection [5]. Here, typically log data is analyzed in order to detect misuses of a system or suspicious events indicating fraud. In particular, financial transactions can be analyzed in order to detect fraudulent accounting [6] and credit card payments logs can be used to detect misused or stolen credit cards [7]. With the strong growth in internet payment systems as well as the increase of offered digital goods, such as ebooks, music, software and movies, fraud detection becomes more and more important in this area. This is due to the fact that pure digital transactions attract scammers since they are less likely to be identified in the real world.

**Data Leakage Prevention** (DLP) is a third important application scenario, where sensitive information is protected by detecting data loss at an early stage [8]. In principle, it is similar to fraud detection, but with a focus on near-real-time analysis such that is serves as a precaution method. In this context, accesses to databases, file servers and other information sources are logged and analyzed in order to detect uncommon access patterns.

In **medical applications** and life sciences, anomaly detection is also utilized. One example is patient monitoring, where electrocardiography (ECG) signals or other body sensors are used to detect critical, possibly life-threatening situations [9]. Additionally, anomaly detection is applied for analyzing medical images, for example computed tomography (CT) in order to detect abnormal cells or tumors. In this application, anomaly detection algorithms rely of course on complex image processing methods as a preprocessing step. In life sciences, anomaly detection might also be utilized to find pathologies and mutants.

Besides these four main application areas, anomaly detection is also used in many **specialized applications**. For example, surveillance camera data can be analyzed for suspicious movements [10], in smart buildings energy consumption anomalies can be found [11], mobile communication networks can be monitored [12] and also forged documents can be detected by a forensic application investigating printed documents [13]. Finally, it is also used in very complex systems in order to detect critical states, of which engineers and developers did not possibly think about during designing the system [14]. Among all these very different application domains, synonyms are often used for the anomaly detection process, which include outlier detection, novelty detection, fraud detection, misuse detection, intrusion detection and behavioral analysis. However, the basic underlying techniques refer to the same algorithms presented in the following sections. More detailed information about application domains as well as overviews of proposed algorithms can be found in the comprehensive surveys of Chandola et al. [15], Hodge et al. [16], Pimentel et al. [17] and Markos et al. [18].

As we can see from this huge variety, also different practical requirements exist for anomaly detection algorithms. In some cases they have to be very fast in a near real-time fashion. In other cases, detection performance is more important due to a high cost of missing an anomaly. In this context, it is also possible to classify the application domains with respect to the point in time when an anomaly should be detected. Among the post-incident analysis and the near real-time detection, additionally a predictive-driven motivation exists, also know as *early warning* [19]. Of course, the latter is the most difficult anomaly detection task, but often major incidents are preceded by minor indications which can be detected.

In this article we present a comparative evaluation of a large variety of anomaly detection algorithms. Clearly, anomaly detection performance is one very important factor for algorithm selection. However, we will also outline strength and weaknesses of the algorithms with respect to their usefulness for specific application scenarios additionally. This supports our main goal that this work should serve as a guideline for selecting an appropriate unsupervised anomaly detection algorithm for a given task.

## Categorization of Anomaly Detection

### Anomaly Detection Setups

**Fig 1:**
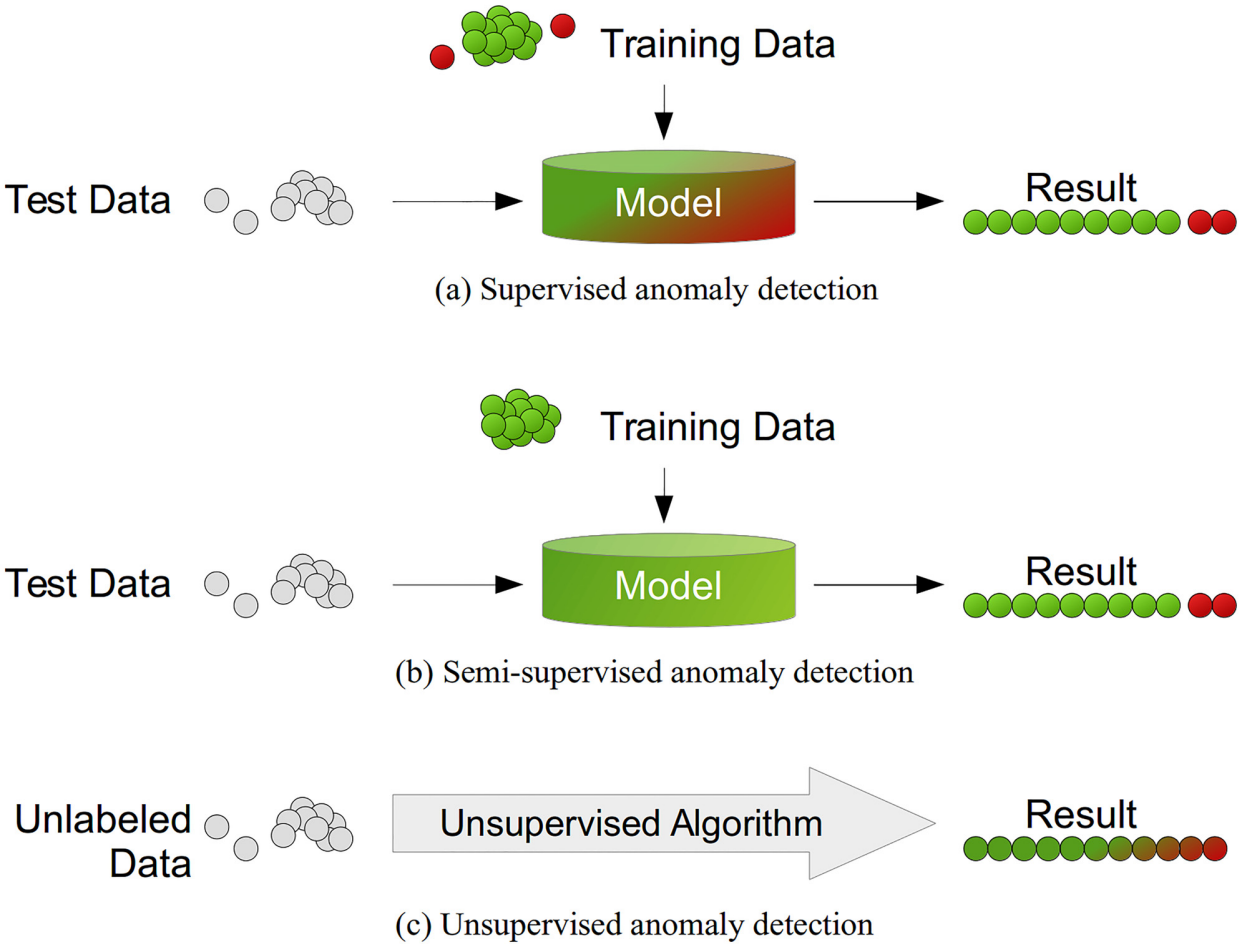
Different anomaly detection modes depending on the availability of labels in the dataset. (a) Supervised anomaly detection uses a fully labeled dataset for training. (b) Semi-supervised anomaly detection uses an anomaly-free training dataset. Afterwards, deviations in the test data from that normal model are used to detect anomalies. (c) Unsupervised anomaly detection algorithms use only intrinsic information of the data in order to detect instances deviating from the majority of the data.

In contrast to the well-known classification setup, where training data is used to train a classifier and test data measures performance afterwards, there are multiple setups possible when talking about anomaly detection. Basically, the anomaly detection setup to be used depends on the labels available in the dataset and we can distinguish between three main types as illustrated in Fig 1:

1. *Supervised Anomaly Detection* describes the setup where the data comprises of fully labeled training and test data sets. An ordinary classifier can be trained first and applied afterwards. This scenario is very similar to traditional pattern recognition with the exception that classes are typically strongly unbalanced. Not all classification algorithms suit therefore perfectly for this task. For example, decision trees like C4.5 [20] cannot deal well with unbalanced data, whereas Support Vector Machines (SVM) [21] or Artificial Neural Networks (ANN) [22] should perform better. However, this setup is practically not very relevant due to the assumption that anomalies are known and labeled correctly. For many applications, anomalies are not known in advance or may occur spontaneously as novelties during the test phase.
2. *Semi-supervised Anomaly Detection* also uses training and test datasets, whereas training data only consists of normal data without any anomalies. The basic idea is, that a model of the normal class is learned and anomalies can be detected afterwards by deviating from that model. This idea is also known as “one-class” classification [23]. Well-known algorithms are One-class SVMs [24] and autoencoders [25]. Of course, in general any density estimation method can be used to model the probability density function of the normal classes, such as Gaussian Mixture Models [26] or Kernel Density Estimation [27].
3. *Unsupervised Anomaly Detection* is the most flexible setup which does not require any labels. Furthermore, there is also no distinction between a training and a test dataset. The idea is that an unsupervised anomaly detection algorithm scores the data solely based on intrinsic properties of the dataset. Typically, distances or densities are used to give an estimation what is normal and what is an outlier. This article only focuses on this unsupervised anomaly detection setup.

### Anomaly Detection Algorithm Output

As an output of an anomaly detection algorithm, two possibilities exist. First, a *label* can be used as a result indicating whether an instance is an anomaly or not. Second, a *score* or confidence value can be a more informative result indicating the degree of abnormality. For supervised anomaly detection, often a label is used due to available classification algorithms. On the other hand, for semi-supervised and unsupervised anomaly detection algorithms, scores are more common. This is mainly due to the practical reasons, where applications often rank anomalies and only report the top anomalies to the user. In this work, we also use scores as output and rank the results such that the ranking can be used for performance evaluation. Of course, a ranking can be converted into a label using an appropriate threshold.

### Types of Anomalies

**Fig 2:**
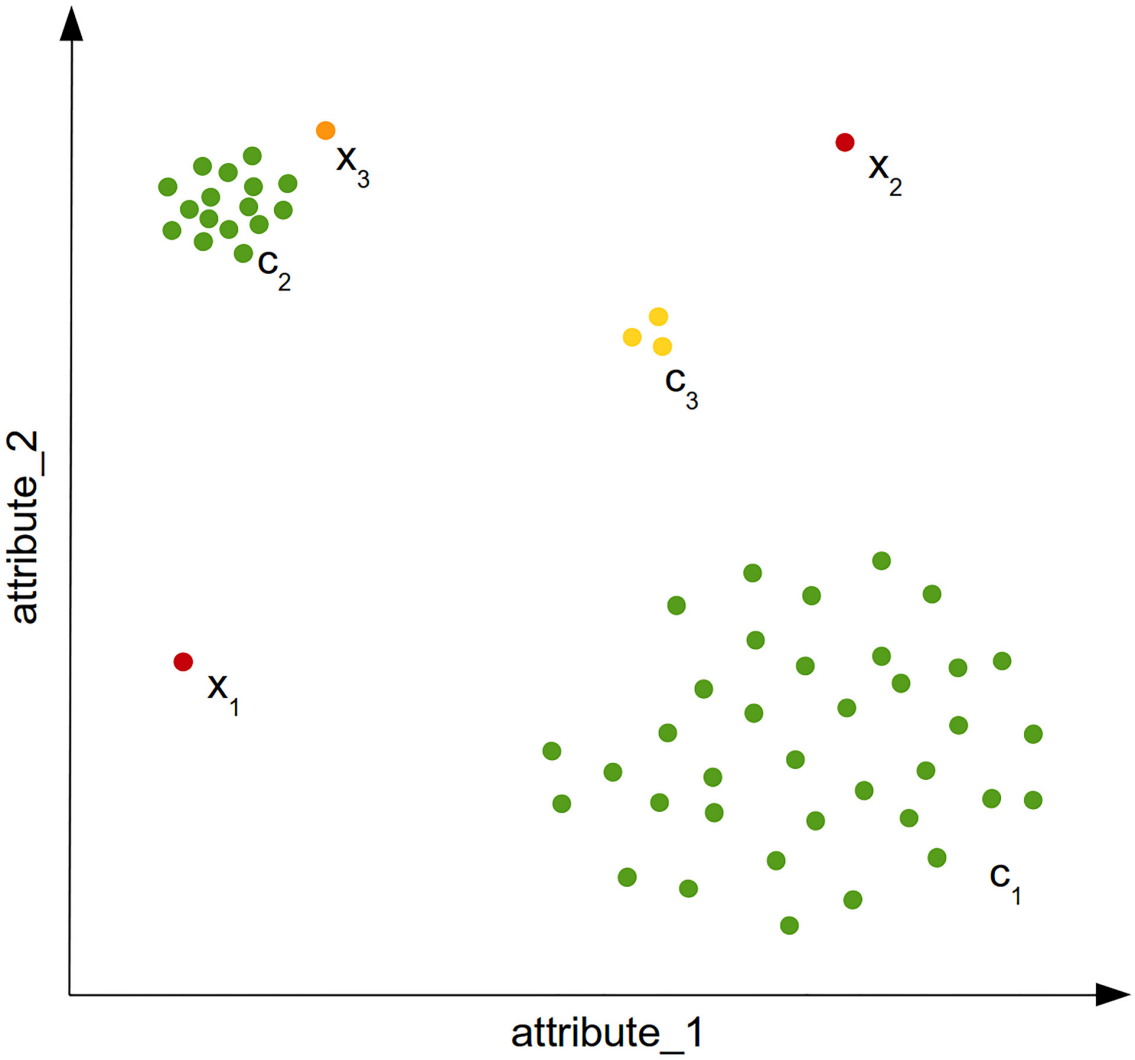
A simple two-dimensional example. It illustrates global anomalies (*x*_1_, *x*_2_), a local anomaly *x*_3_ and a micro-cluster *c*_3_.

The main idea of unsupervised anomaly detection algorithms is to detect data instances in a dataset, which deviate from the norm. However, there are a variety of cases in practice where this basic assumption is ambiguous. Fig 2 illustrates some of these cases using a simple two-dimensional dataset. Two anomalies can be easily identified by eye: *x*_1_ and *x*_2_ are very different from the dense areas with respect to their attributes and are therefore called *global anomalies*. When looking at the dataset globally, *x*_3_ can be seen as a normal record since it is not too far away from the cluster *c*_2_. However, when we focus only on the cluster *c*_2_ and compare it with *x*_3_ while neglecting all the other instances, it can be seen as an anomaly. Therefore, *x*_3_ is called a *local anomaly*, since it is only anomalous when compared with its close-by neighborhood. It depends on the application, whether local anomalies are of interest or not. Another interesting question is whether the instances of the cluster *c*_3_ should be seen as three anomalies or as a (small) regular cluster. These phenomena is called *micro cluster* and anomaly detection algorithms should assign scores to its members larger than the normal instances, but smaller values than the obvious anomalies. This simple example already illustrates that anomalies are not always obvious and a score is much more useful than a binary label assignment.

To this end, an anomaly is always referred to a single instance in a dataset only occurring rarely. In reality, this is often not true. For example, in intrusion detection, anomalies are often referred to many (suspicious) access patterns, which may be observed at a larger amount as the normal accesses. In this case, an unsupervised anomaly detection algorithm directly applied on the raw data will fail. The task of detecting single anomalous instances in a larger dataset (as introduced so far) is called *point anomaly detection* [15]. Nearly all available unsupervised anomaly detection algorithms today are from this type. If an anomalous situation is represented as a set of many instances, this is called a *collective anomaly*. Each of these instances is not necessarily a point anomaly, but only a specific combination thereof defines the anomaly. The previous given example of occurring multiple specific access patterns in intrusion detection is such a collective anomaly. A third kind are *contextual anomalies*, which describe the effect that a point can be seen as normal, but when a given context is taken into account, the point turns out to be an anomaly. The most commonly occurring context is time. As an example, suppose we measure temperature in a range of 0°to 35°C during the year. Thus, a temperature of 26°C seems pretty normal, but when we take the context time into account (e.g. the month), such a high temperature of 26°C during winter would definitively be considered as an anomaly.

Fortunately, it is still possible to utilize point anomaly detection algorithms to detect contextual and collective anomalies. In order to do so, the context itself can be included as a new feature. Concerning our simple example of the temperature measurement, a direct inclusion of the month as a second feature is easily possible. However, in more complex scenarios, one or more newly derived features might be required to transform the contextual anomaly detection task into a point anomaly detection problem. For addressing the collective anomalies, correlation, aggregation and grouping is used to generate a new dataset with a different representation of the features [11]. This transformation from a collective anomaly detection task to a point anomaly detection task requires a solid background knowledge of the dataset and often results in a point anomaly detection dataset, which features and instances are very different from the original raw data. This semantic transformation is also called the generation of a *data view* [28]. As we can conclude from these three different type of anomalies, not every anomaly detection task is suitable to be processed directly using an anomaly detection algorithm. In fact, many practical anomaly detection problems often require a preprocessing in order to generate the appropriate data views. In this work, we also carefully selected the datasets to be point anomaly detection problems, such that no further preprocessing is necessary and we can directly compare the detection performance of the different algorithms.

### Normalization

When a dataset was preprocessed such that it represents a point anomaly detection problem, the final step before the unsupervised anomaly detection algorithm can be applied is normalization. Similar to the generation of the data view, normalization should also be performed with taking background knowledge into account. Typical normalization methods are min-max normalization, where every feature is normalized into a common interval (for example [0, 1]), and standardizing, where each feature is transformed such that its mean is zero and its standard deviation is one. In practical applications, the min-max normalization is often used, so do we in the evaluation in this article. Please note, that sometimes straight-forward normalization can also be contra-productive. Let’s assume we have a categorical binary feature converted to [0, 1] and a numerical value measuring a length normalized to [0, 1]. Since the categorical binary feature results in distances being either one or zero, it has a much bigger influence on the result as the numerical value. This is the reason, why background information is also important during normalization to avoid these errors in the normalization process. Possible solutions to that challenge include using different intervals for the different semantic features, or when categorical features come into play, using a weighted distance function [29].

## Related Work

It could already be inferred from the previous sections that this article primarily deals with multivariate tabular data. Differently structured data, such as graphs or sequential data, is often processed in machine learning using dedicated algorithms. This also holds true in anomaly detection and there exist many algorithms for detecting anomalies in graphs [30], in sequences and time series [31] and for addressing spatial data [32]. However, these specialized algorithms are not evaluated in this work, which focuses on tabular data.

Not many comparative studies on unsupervised anomaly detection do exist today. On the one hand, authors of newly proposed algorithms compare their results with state-of-the-art techniques, for example LOF and *k*-NN, but often datasets are not published and the evaluation lacks in some other evaluation criteria (local vs. global or parameter *k*). On the other hand, some studies have been published referring to a specific application scenario, often with a single dataset only. Lazarevic et al. [33] compared LOF, *k*-NN, PCA and unsupervised SVM for intrusion detection using the KDD-Cup99 dataset. A similar study by Eskin et al. [34] evaluates clustering, *k*-NN as well as a one-class SVM on the same dataset. A broader study using six different methods for unsupervised anomaly detection was performed by NASA [14] for detecting engine failures of space shuttles. Unfortunately, the dataset is not available and the algorithms used are besides GMM and one-class SVMs four commercially available software systems. Auslander et al. [35] applied *k*-NN, LOF and clustering on maritime video surveillance data. Ding et al. [36] studied SVDD, a *k*-NN classifier, *k*-means and a GMM for detecting anomalies in ten different datasets. Although the authors claim to evaluate unsupervised techniques, the use of a training phase indicates a semi-supervised setup to our understanding. Carrasquilla [37] published a study on comparing different anomaly detection algorithms based on 10 different datasets. Unfortunately, only one unsupervised anomaly detection algorithm was applied, whereas its results were compared to other supervised anomaly detection algorithms. Some of the datasets used in this study are also used as a basis in our evaluation, but with an appropriate preprocessing. All related work concerning the particular algorithms used in this study can be found in the next section.

Besides studies evaluating a single algorithm only, outlier ensembles [38, 39] is a technique of combining multiple unsupervised anomaly detection algorithms in order to boost their joint anomaly detection performance. Since unsupervised anomaly detection does not rely on labeled data, this task is very challenging and often restricted to simple combinations. In this article we do not evaluate ensembles, although the results reported here might serve as a selection criteria for these algorithms in this currently evolving new research field.

## Unsupervised Anomaly Detection Algorithms

**Fig 3:**
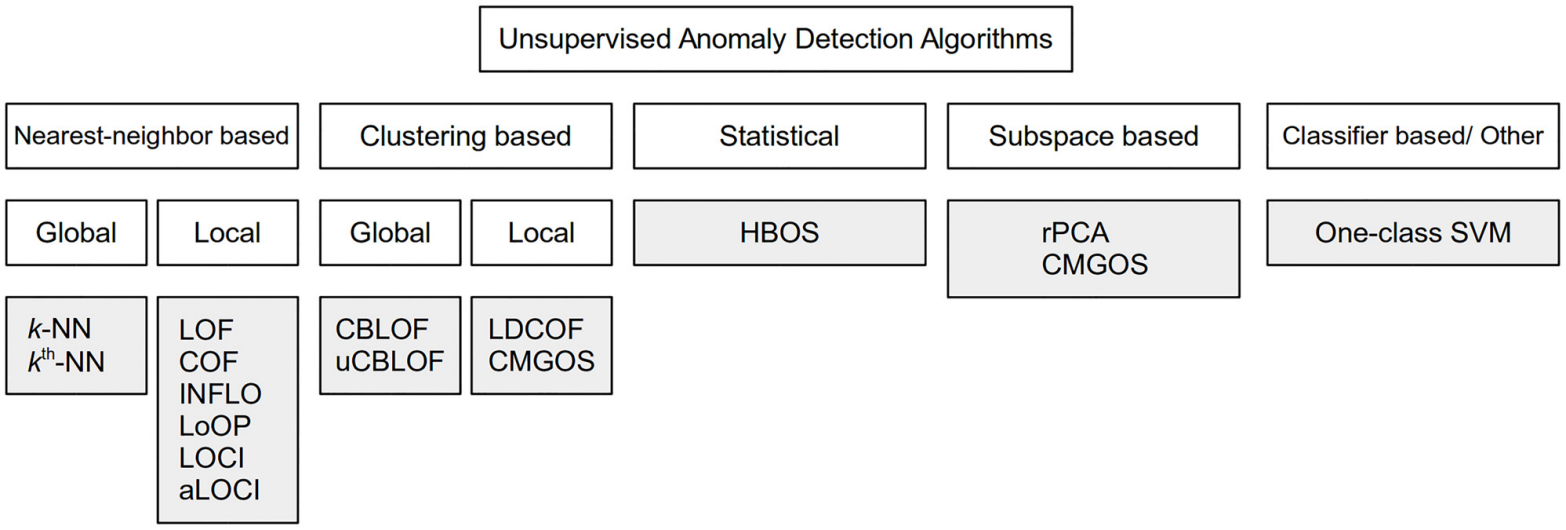
A taxonomy of unsupervised anomaly detection algorithms comprising of four main groups. Note that CMGOS can be categorized in two groups: It is a clustering-based algorithm as well as estimating a subspace of each cluster.

Unsupervised anomaly detection algorithms can be roughly categorized into the following main groups [15] as illustrated in Fig 3: (1) Nearest-neighbor based techniques, (2) Clustering-based methods and (3) Statistical algorithms. Recently, also a new group is emerging based on (4) Subspace techniques. In this work, we cover all of these categories with a focus on nearest-neighbor and clustering-based anomaly detection, the by far most used categories in practice. Furthermore, other algorithms exist, which are not direct members of these categories, often based on available classification algorithms such as neural networks [25] or support vector machines [40]. It is not an easy task to select a proper subset for this work keeping in mind that dozens of different algorithms have been proposed. However, our selection is based on practical applications in the past and attention in the scientific community. Since one goal of this work is also to standardize datasets, we also welcome other researchers to compare their proposed methods with the results presented here. In this section, we shortly introduce the algorithms and their main ideas, but due to the high number of algorithms, the interested reader is referred to the authors original publication for more details.

### *k*-NN Global Anomaly Detection

The *k*-nearest-neighbor global unsupervised anomaly detection algorithm is a straightforward way for detecting anomalies and not to be confused with *k*-nearest neighbor classification. As the name already implies, it focuses on global anomalies and is not able to detect local anomalies. First, for every record in the dataset, the *k*-nearest-neighbors have to be found. Then, an anomaly score is computed using these neighbors, whereas two possibilities have been proposed: Either the distance to the *k^th^*-nearest-neighbor is used (a single one) [41] or the average distance to all of the *k*-nearest-neighbors [42] is computed. In the following, we refer to the first method as *k^th^*-NN and the latter as *k*-NN. In practical applications, the *k*-NN method is often preferred [13, 19]. However, the absolute value of the score depends very much on the dataset itself, the number of dimensions, and on normalization. As a result, it is in practice not easy to select an appropriate threshold, if required.

The choice of the parameter *k* is of course important for the results. If it is chosen too low, the density estimation for the records might be not reliable. On the other hand, if it is too large, density estimation may be too coarse. As a rule of thumb, *k* should be in the range 10 < *k* < 50. In classification, it is possible to determine a suitable *k*, for example by using cross-validation. Unfortunately, there is no such technique in unsupervised anomaly detection due to missing labels. For that reason, we use later in the evaluation many different values for *k* and average in order to get a fair evaluation when comparing algorithms.

**Fig 4:**
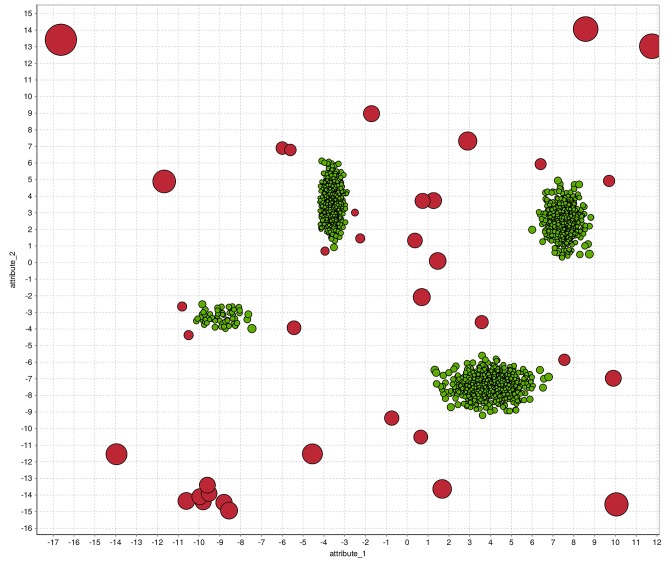
A visualization of the results of the *k*-NN global anomaly detection algorithm. The anomaly score is represented by the bubble size whereas the color shows the labels of the artificially generated dataset.

In Fig 4 we exemplary illustrate how the result of an unsupervised anomaly detection algorithm (here: *k*-NN with *k* = 10) can be visualized. The plot was generated using a simple, artificially generated two-dimensional dataset with four Gaussian clusters and uniformly sampled anomalies. After applying the global *k*-NN, the outlier scores are visualized by the bubble-size of the corresponding instance. The color indicates the label, whereas anomalies are red. It can be seen, that *k*-NN cannot detect the anomalies close to the clusters well and assign small scores.

### Local Outlier Factor (LOF)

The local outlier factor [43] is the most well-known local anomaly detection algorithm and also introduced the idea of local anomalies first. Today, its idea is carried out in many nearest-neighbor based algorithms, such as in the ones described below. To calculate the LOF score, three steps have to be computed:

1. The *k*-nearest-neighbors have to be found for each record *x*. In case of distance tie of the *k^th^* neighbor, more than *k* neighbors are used.
2. Using these *k*-nearest-neighbors *N_k_*, the local density for a record is estimated by computing the local reachability density (LRD):
  $$\begin{array}{c} LRD_{k}\left(x\right)=1/\left(\frac{\sum_{o\in N_{k}\left(x\right)}d_{k}\left(x,o\right)}{\left|N_{k}\left(x\right)\right|}\right) \end{array}$$ (1)
  whereas *d_k_*(·) is the reachability distance. Except for some very rare situations in highly dense clusters, this is the Euclidean distance.
3. Finally, the LOF score is computed by comparing the LRD of a record with the LRDs of its *k* neighbors:
  $$\begin{array}{c} LOF\left(x\right)=\frac{\sum_{o\in N_{k}\left(x\right)}\frac{LRD_{k}\left(o\right)}{LRD_{k}\left(x\right)}}{\left|N_{k}\left(x\right)\right|} \end{array}$$ (2)

The LOF score is thus basically a ratio of local densities. This results in the nice property of LOF, that normal instances, which densities are as big as the densities of their neighbors, get a score of about 1.0. Anomalies, which have a low local density, will result in larger scores. At this point it is also clear why this algorithm is local: It only relies on its direct neighborhood and the score is a ratio mainly based on the *k* neighbors only. Of course, global anomalies can also be detected since they also have a low LRD when comparing with their neighbors. It is important to note that in anomaly detection tasks, where local anomalies are not of interest, this algorithm will generate a lot of false alarms as we found out during our evaluation. Again, the setting of *k* is crucial for this algorithm. Besides trying out different values for *k*, the authors of the algorithm suggested to use an ensemble strategy for computing the LOF [43]. Here, scores for different *k*’s up to an upper bound are computed and then, the maximum of these scores is taken. Besides computing the LOF score for a single *k*, we also take this strategy into account in our evaluation, referring to it as LOF-UB (upper bound). For comparison reasons, we also use different upper bounds and average the results again.

### Connectivity-Based Outlier Factor (COF)

**Fig 5:**
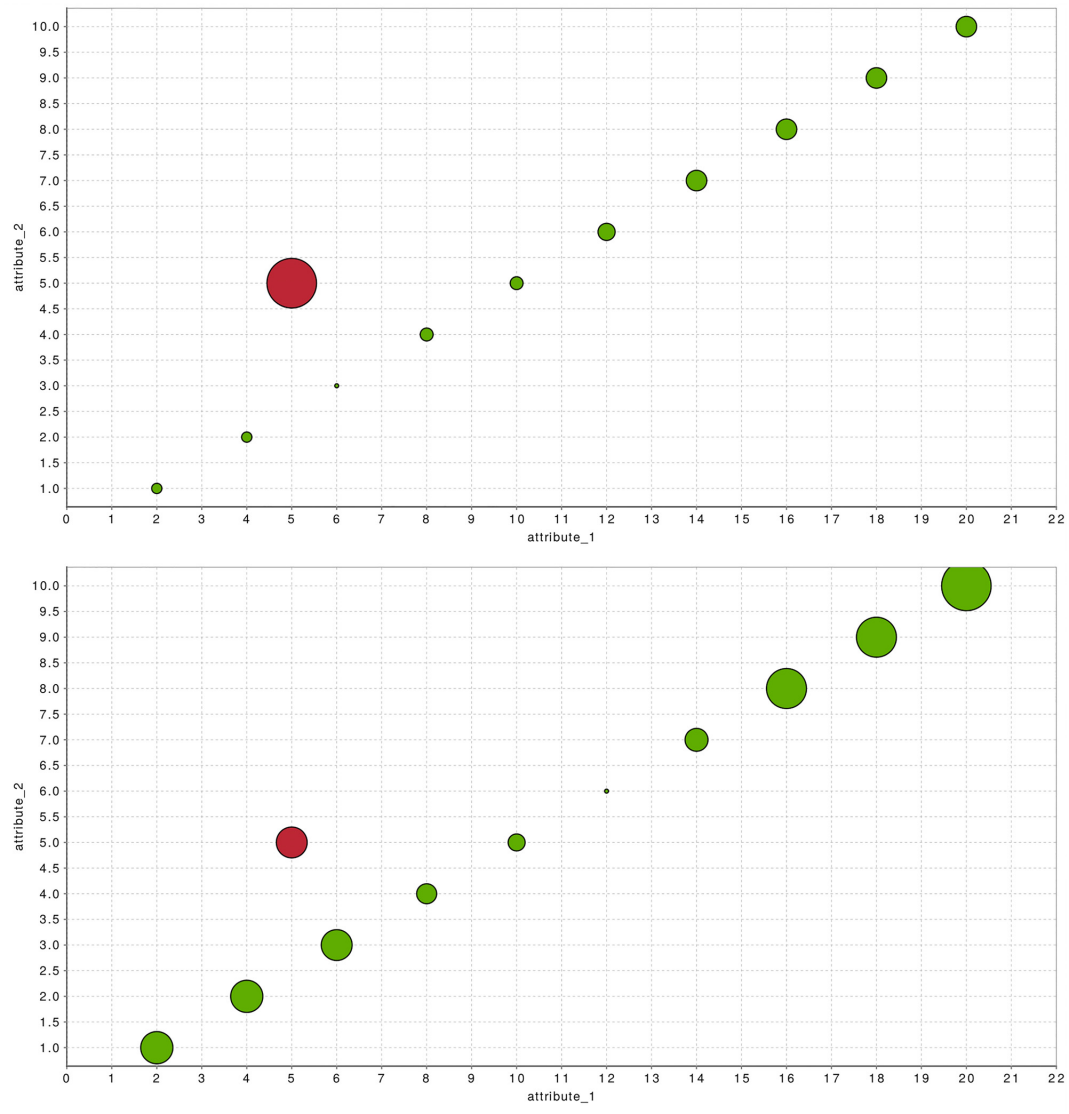
Comparing COF (top) with LOF (bottom) using a simple dataset with a linear correlation of two attributes. It can be seen that the spherical density estimation of LOF fails to recognize the anomaly, whereas COF detects the non-linear anomaly (*k* = 4).

The connectivity-based outlier factor [44] is similar to LOF, but the density estimation for the records is performed differently. In LOF, the *k*-nearest-neighbors are selected based on the Euclidean distance. This indirectly assumes, that the data is distributed in a spherical way around the instance. If this assumption is violated, for example if features have a direct linear correlation, the density estimation is incorrect. COF wants to compensate this shortcoming and estimates the local density of the neighborhood using an shortest-path approach, called the chaining distance. Mathematically, this chaining distance is the minimum of the sum of all distances connecting all *k* neighbors and the instance. For simple examples, where features are obviously correlated, this density estimation approach performs much more accurate [29]. Fig 5 shows the outcome for LOF and COF in direct comparison for a simple two-dimensional dataset, where the attributes have a linear dependency. It can be seen that the spherical density estimation of LOF cannot detect the outlier, but COF succeeded by connecting the normal records with each other for estimating the local density.

### Influenced Outlierness (INFLO)

**Fig 6:**
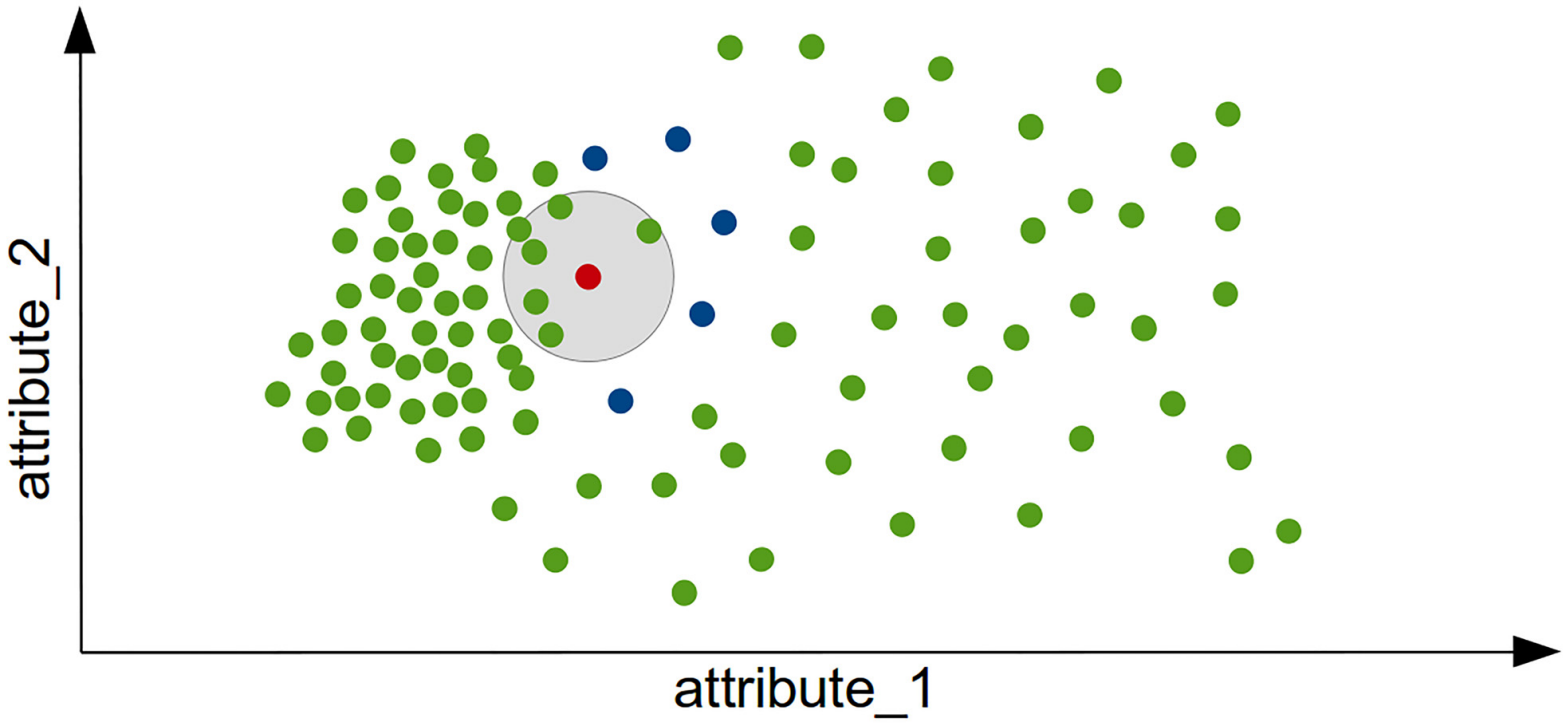
Comparing INFLO with LOF shows the usefulness of the reverse neighborhood set. For the red instance, LOF takes only the neighbors in the gray area into account resulting in a high anomaly score. INFLO additionally takes the blue instances into account (reverse neighbors) and thus scores the red instance more normal.

When a dataset contains clusters with different densities and they are close to each other, it can be shown that LOF fails scoring the instances at the borders of the clusters correctly. The influenced outlierness (INFLO) [45] algorithm uses besides the *k*-nearest-neighbors additionally a reverse nearest neighborhood set, in which records are stored for with the current record is a neighbor. For computing the INFLO score, both neighborhood sets are combined. Then, the local density of this set and the score is computed the same way as for LOF. This procedure is illustrated in Fig 6, where for the red instance the 6-nearest-neighbors reside in the gray area. This red instance will clearly be detected as an anomaly by LOF, since five of its neighbors have a much higher local density. For INFLO, also the instances are taken into account for which the red instance is a neighbor (the blue instances). Using this extended set, the red instance is less likely to be detected as an anomaly by INFLO. Please note, that the set of *k*-nearest-neighbors typically contains *k* instances (with the exception of ties), whereas the reverse nearest neighborhood set may contain any amount. Depending on the data, it might contain no instance, exactly *k* or even more instances. When using this strategy, it is possible to compute more accurate anomaly scores when clusters of different densities are close to each other.

### Local Outlier Probability (LoOP)

Until now, all presented algorithms output anomaly scores, which are more handy than binary labels. When comparing the global *k*-NN algorithm and LOF, the property of having a reference point for normal instances of LOF seems even better than the arbitrary score of *k*-NN. Unfortunately, it is still not clear in LOF, above which score threshold we can clearly think about an anomaly. The local outlier probability (LoOP) [46] tries to address this issue by outputting an anomaly probability instead of a score, which might also result in better comparison of anomalous records between different datasets.

Similar to the previous local algorithms, LoOP also uses a neighborhood set for local density estimation. In contrast to other algorithms, it computes this density differently: The basic assumption is that the distances to the nearest neighbors follow a Gaussian distribution. Since distances are always positive, LoOP assumes a “half-Gaussian” distribution and uses its standard deviation, called the probabilistic set distance. It is used (similar to LOF) as a local density estimation—the ratios of each instance compared to its neighbors results in a local anomaly detection score. For converting this score into a probability, a normalization and a Gaussian error function is applied finally. The idea of having a probabilistic output instead of a score is very useful. However, some critical thoughts should arise in this context [29]. For example, if the algorithm assigns a 100% probability to an instance, what would happen, if we add another instance to the dataset which is more anomalous then that? As we can see from this simple example, probabilities are still relative to the data and might not differ too much from a normalized score.

### Local Correlation Integral (LOCI)

For all of the above algorithms, choosing *k* is a crucial decision for detection performance. As already mentioned, there is no way of estimating a good *k* based on the data. Nevertheless, the local correlation integral (LOCI) [47] algorithm addresses this issue by using a maximization approach. The basic idea is that all possible values of *k* are used for each record and finally the maximum score is taken. To achieve this goal, LOCI defines the *r*-neighborhood by using a radius *r*, which is expanded over time. Similar to LoOP, the local density is also estimated by using a half-Gaussian distribution, but here the amount of records in the neighborhood is used instead of the distances. Also, the local density estimation is different in LOCI: It compares two different sized neighborhoods instead of the ratio of the local densities. A parameter *α* controls the ratio of the different neighborhoods. Removing the critical parameter *k* comes at a price. Typically, nearest-neighbor based anomaly detection algorithms have computational complexity of *O*(*n*^2^) for finding the nearest neighbors. Since in LOCI additionally the radius *r* needs to be expanded from one instance to the furthest, the complexity increases to *O*(*n*^3^), which makes LOCI too slow for larger datasets.

### Approximate Local Correlation Integral (aLOCI)

The authors of LOCI were aware of the long runtime and proposed aLOCI [48], a faster but approximate version of LOCI. aLOCI uses quad trees to speed up the counting of the two neighborhoods using some constraints for *α*. If a record is in the center of a cell of such a quad tree, the counting estimation is good, but if it is at the border, the approximation might be bad. For that reason, multiple (*g*) quad trees are constructed with the hope, that there is a good approximative tree for every instance. Furthermore, the tree depth (*L*) needs to be specified. The authors claim that the total complexity of their algorithm, comprising of tree creation and scoring, is *O*(*NLdg* + *NL*(*dg* + 2^*d*^)), whereas *d* is the number of dimensions. As typical for tree approaches, it can be seen that the number of dimensions has a very negative impact on the runtime. During our evaluation, we experienced very different results from aLOCI. Sometimes results seem reasonable and sometimes results showed a very poor anomaly detection performance. This observation was tracked down the tree creation process. For a perfect estimation, *N* trees are required. Since the trick of this algorithm is to use only *g* trees, this also turned out to be a weak point: If, by chance, the trees represented the normal instances well, many approximations were correct and thus the output of the algorithm. On the other hand, if the trees did not well represent the majority of the data, the anomaly detection performance was unacceptable.

### Cluster-Based Local Outlier Factor (CBLOF/ uCBLOF)

**Fig 7:**
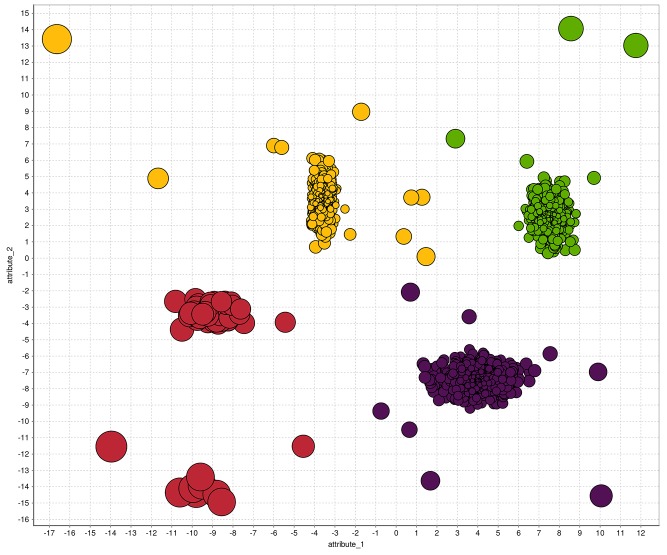
A visualization of the results for the uCBLOF algorithm. The anomaly score is represented by the bubble size, whereas the color corresponds to the clustering result of the preceded *k*-means clustering algorithm. Local anomalies are obviously not detected using uCBLOF.

All previous anomaly detection algorithms are based on density estimation using nearest-neighbors. In contrast, the cluster-based local outlier factor (CBLOF) [49] uses clustering in order to determine dense areas in the data and performs a density estimation for each cluster afterwards. In theory, every clustering algorithm can be used to cluster the data in a first step. However, in practice *k*-means is commonly used to take advantage of the low computational complexity, which is linear compared to the quadratic complexity of the nearest-neighbor search. After clustering, CBLOF uses a heuristic to classify the resulting clusters into large and small clusters. Finally, an anomaly score is computed by the distance of each instance to its cluster center multiplied by the instances belonging to its cluster. For small clusters, the distance to the closest large cluster is used. The procedure of using the amount of cluster members as a scaling factor should estimate the local density of the clusters as stated by the authors. We showed in previous work that this assumption is not true [50] and might even result in a incorrect density estimation. Therefore, we additionally evaluate a modified version of CBLOF which simply neglects the weighting, referred to as unweighted-CBLOF (uCBLOF) in the following. The results of uCBLOF using a simple two-dimensional dataset are visualized in Fig 7, where the color corresponds to the clustering result of the preceding *k*-means clustering algorithm. Similar to the nearest-neighbor based algorithms, the number of initial clusters *k* is also a critical parameter. Here, we follow the same strategy as for the nearest-neighbor based algorithms and evaluate many different *k* values. Furthermore, *k*-means clustering is a non-deterministic algorithm and thus the resulting anomaly scores can be different on multiple runs. To this end we follow a common strategy, which is to apply *k*-means many times on the data and pick the most stable result. However, clustering-based anomaly detection algorithms are very sensitive to the parameter *k*, since adding just a single additional centroid might lead to a very different outcome.

### Local Density Cluster-based Outlier Factor (LDCOF)

As already pointed out, the local density estimation of CBLOF using only the amount of cluster members is controversial. Our extension uCBLOF is in fact not a local anomaly detection method any more since the density of the cluster is completely neglected. The local density cluster-based outlier factor LDCOF [50] addresses this shortcoming by estimating the clusters’ densities assuming a spherical distribution of the cluster members. Similar to CBLOF, *k*-means clustering is performed first as well as the following procedure of separating the clusters into small and large clusters. Then, for each cluster, the average distance of all cluster members to the centroid is calculated. Finally, the LDCOF score is computed by dividing the distance of an instance to its cluster center by the average distance. The result of this procedure is that the LDCOF score is a local score with respect to the possibly varying cluster densities. One advantage of LDCOF is also that the score has some relative reference point, similar to LOF: A score of 1.0 or below will be assigned to normal instances.

### Clustering-based Multivariate Gaussian Outlier Score (CMGOS)

The clustering-based multivariate Gaussian outlier score is another enhancement of cluster-based anomaly detection [29]. In CMGOS, the local density estimation is performed by estimating a multivariate Gaussian model, whereas the Mahalanobis distance [51] serves as a basis for computing the anomaly score. As in the previously introduced algorithms, a *k*-means clustering and the separation into small and large clusters is performed first. Then, for each cluster, the covariance matrix Σ is computed robustly. Finally, the CMGOS score is computed by dividing the Mahalanobis distance of an instance to its nearest cluster center by the chi-squared distribution with a certain confidence interval. The latter serves again as a normalization step such that outlier scores of 1.0 or below indicate a high probability of the instance to be normal. Due to the nature of the Mahalanobis distance, scores of outliers increase quickly, such that in practical applications extraordinary large scores can be observed (compared to other methods). For the estimation of the covariance matrix, robustness to outliers is essential since outliers are known to have a significant impact on the variance. In total, three different estimation methods are proposed: (1) Reduction. Here, the covariance is computed twice. After a first iteration, outliers are removed and covariance is computed again. This procedure is similar to the well-known univariate Grubb’s Test [1]. (2) Regularization. This approach follows an idea from classification [52], where the covariance matrix is a weighted sum of the covariance matrix of the cluster and the global covariance matrix. (3) Minimum Covariance Determinant (MCD) [53]. This compute intense approach follows the idea to estimate a compact covariance matrix by a brute-force search for normal records, which is done by minimizing the determinant. Our evaluation is based on an approximative alternative [54], since the brute-force search is not suitable for large datasets. We refer in our evaluation to the methods as CMGOS-Red, CMGOS-Reg and CMGOS-MCD, respectively.

### Histogram-based Outlier Score (HBOS)

The histogram-based outlier score [55] is a simple statistical anomaly detection algorithm assuming independence of the features. The basic idea is, that for each feature of the dataset, a histogram is created. Then, for each instance in the dataset, the inverse height of the bins it resides (representing the density estimation) of all features are multiplied. The idea is very similar to the Naive Bayes algorithm in classification, were all independent feature probabilities are multiplied. The idea of using histograms for fast semi-supervised anomaly detection is also very popular in intrusion detection, were a histogram of normal data is learned [56]. On the first sight, it might seem a bit contra-productive to neglect the dependencies among features. However, this comes with a big advantage, which is processing speed. HBOS can process a dataset under a minute, whereas nearest-neighbor based computations take over 23 hours [29]. As a further remark, the drawback of assuming feature independence becomes less severe when a dataset has a high number of dimensions due to a larger sparsity. As a critical parameter, the number of bins *k* needs to be defined. Furthermore, HBOS allows two different histogram creation modes: (1) Static bin sizes with a fixed bin width and (2) dynamic bins such that the number of bins is approximately the same. The latter results in a histogram with different bin widths, but it can still be used for density estimation using the area of a bin. The advantage of the second histogram creation technique is, that the density estimation is more robust in case of having large outlying values. In our evaluation, we use this second “dynamic bin width” mode as well as different settings for *k*.

### One-Class Support Vector Machine

One-class support vector machines [24] are often used for semi-supervised anomaly detection [15]. In this setting, a one-class SVM is trained on anomaly-free data and later, the SVM classifies anomalies and normal data in the test set. One-class SVMs intend to separate the origin from the data instances in the kernel space, which results in some kind of complex hulls describing the normal data in the feature space. Although one-class SVMs are heavily used as a semi-supervised anomaly detection method, it is an unsupervised algorithm by design when using a soft-margin. In particular, it has been shown that they converge to the true density level set [57]. In the unsupervised anomaly detection scenario, the one-class SVM is trained using the dataset and afterwards, each instance in the dataset is scored by a normalized distance to the determined decision boundary [40]. The parameter *ν* needs to be set to a value lager than zero such that the contained anomalies are correctly handled by a soft-margin. Additionally, one-class SVMs have been modified such that they include further robust techniques for explicitly dealing with outliers during training [40]. The basic idea is that anomalies contribute less to the decision boundary as normal instances. Two different techniques were developed, whereas the *η* one-class SVM showed superior results. In this enhancement, *η* is a further optimization objective during training, which estimates the normality of an instance. In our evaluation we are using both unsupervised algorithms, the regular one-class SVM as well as the extended *η* one-class SVM.

### Robust Principal Component Analysis (rPCA)

Principal component analysis is a commonly used technique for detecting subspaces in datasets. It also may serve an an anomaly detection technique, such that deviations from the normal subspaces may indicate anomalous instances. The principal components are the eigenvectors of the covariance matrix and thus their computation shares the same difficulties as CMGOS: Anomalies have a big influence on the covariance matrix and density estimation might be incorrect. Almost identical to the reduction technique of CMGOS, a robust version was proposed [58], which also computes the covariance matrix twice based on the Mahalanobis distance. Once the principal components are determined, the question is which components should be used to score anomalous instances. Using the major components show global deviations from the majority of the data whereas using minor components can indicate smaller local deviations. In our evaluation we follow different strategies, namely using all components, using major components only, using minor components only and finally using major and minor components while neglecting the ones in the middle [59]. Please note that there is a strong connection of rPCA and CMGOS-Red: When rPCA takes all components into consideration, the method is basically the same as applying CMGOS with setting *k* = 1.

### Reviewing Algorithm Complexities and Implementation

Concerning the nearest-neighbor based algorithms with the exception of LOCI, the computational complexity of finding the nearest-neighbors is *O*(*n*^2^). The remaining computations, for example the density or LOF calculations, can be neglected in practice (less than 1% of runtime). Thus, all of these algorithms perform similar in terms of runtime. In our implementation, we used many optimizations so that the algorithms still perform well on large-scale datasets. In particular, first duplicates are removed from the data and a weight matrix is stored. This procedure might reduce the dataset size and thus save computation time. Memory consumption was reduced by storing only the top-*k*-neighbors during the search. Additionally, a smart parallelization technique was implemented depending on the number of dimensions. More information about the enhancements can be found in [50]. For LOCI, the computational complexity is *O*(*n*^3^) and the memory complexity is *O*(*n*^2^), which makes the algorithm practically too demanding for real-world applications. aLOCI on the contrary is faster and the runtime depends on the number of quad-trees to be used.

Concerning the cluster-based methods (except for CMGOS-MCD), the main computational complexity is due to the clustering algorithm, which is typically faster than *O*(*n*^2^) if *k*-means is applied. In practice, when *k*-means is run several times in order to get stable clustering result, the runtime advance is reduced but still present. For large-scale datasets and big data, clustering-based methods have thus a performance advance compared to nearest-neighbor based methods. However, CMGOS-MCD is an exception here since the MCD computation is again quadratic for each cluster. Furthermore, as already mentioned, HBOS is even faster than the clustering-based algorithms and a good candidate for near real-time large-scale applications.

The complexity of the one-class SVM based algorithms is hard to determine since it depends on the number of support vectors and thus on the structure of the data. Furthermore, the applied gamma tuning of the SVMs has a huge impact on runtime since its computation has a quadratic complexity. Lastly, the complexity of rPCA is *O*(*d*^2^ *n* + *d*^3^) and therefore depends heavily on the number of dimensions. If the number of dimensions is small, the algorithm competes in practice among the fastest algorithms in our trials. The source code of the algorithms and our optimizations are published as an open source plug-in (available at http://git.io/vnD6n) of the RapidMiner [60] data mining software.

## Datasets for Benchmarking

Although unsupervised anomaly detection does not utilize any label information in practice, they are needed for evaluation and comparison. When new algorithms are proposed, it is common practice that an available public classification dataset is modified and the method is compared with the most known algorithms such as *k*-NN and LOF. There is a set of typically used datasets for classification, which are retrieved from UCI machine learning repository [61]. The typical preprocessing comprises of selecting one class as the anomalous class and sub-sampling some small amount of instances from that randomly. Unfortunately, the resulting datasets are hardly published and cannot be regenerated by other scientists. Since the number of anomalies is typically very low, a different subset might result in very different detection scores. To this end, we only found three different datasets available online [62, 63]. With this work we want to compensate this shortcoming and present and publish more different meaningful datasets, such that algorithms are better comparable in the future. Please note that some of the datasets have already been introduced previously in the first author’s Ph.D. Thesis [29] We also put a strong emphasis on a semantic background such the evaluation makes sense. This includes the two assumptions that (1) anomalies are rare and (2) are different from the norm. Additionally, we consider only point anomaly detection tasks as meaningful datasets for benchmarking since a different preprocessing might again lead to non-comparable results. For example, the dataset Poker Hand, which was used for evaluation before [37], is not used because the anomalies (special winning card decks) violate the assumption (2). Similarly, the use of the very popular KDD-Cup99 dataset needs special attention, which was originally used for benchmarking intrusion detection classification systems. Many attacks (anomalies) in the dataset define a collective anomaly detection problem and can thus not be used. Since the dataset is so popular, a point anomaly detection task was extracted as stated below.

If the following, we describe the datasets and our preprocessing in more detail. All modifications have been made publicly available (http://dx.doi.org/10.7910/DVN/OPQMVF). A summary about the resulting dataset characteristics is given below in Table 1.

**Table 1:** The 10 datasets used for comparative evaluation of the unsupervised anomaly detection algorithms from different application domains. A broad spectrum of size, dimensionality and anomaly percentage is covered. They also differ in difficulty and cover local and global anomaly detection tasks.

Dataset	Size	Dimensions	Outliers	Percentage
b-cancer	367	30	10	2.72
pen-global	809	16	90	11.1
letter	1,600	32	100	6.25
speech	3,686	400	61	1.65
satellite	5,100	36	75	1.49
pen-local	6,724	16	10	0.15
annthyroid	6,916	21	250	3.61
shuttle	46,464	9	878	1.89
aloi	50,000	27	1508	3.02
kdd99	620,098	38	1,052	0.17

**Breast Cancer Wisconsin (Diagnostic)** The features of the *breast-cancer* dataset are extracted from medical images of a fine needle aspirate (FNA) describing the cell nuclei [64]. The task of the UCI dataset is to separate cancer from healthy patients. From the 212 malignant instances, we kept the first 10 as anomalies (similar to [46]). This results in a unsupervised anomaly detection dataset containing 367 instances in total and having 2.72% anomalies.

**Pen-Based Recognition of Handwritten Text (global)** This UCI dataset contains the handwritten digits 0–9 of 45 different writers, which we will use twice. Here, in the “global” task, we only keep the digit 8 as the normal class and sample the 10 digits from all of the other classes as anomalies. This results in one big normal cluster and global anomalies sparsely distributed. The resulting *pen-global* dataset has 16 dimensions and 809 instances including a large amount of anomalies (11.1%).

**Pen-Based Recognition of Handwritten Text (local)** The previous dataset is used again, but now with a different preprocessing. All digits are kept, except for the digit 4. From this class, the first 10 instances are kept (similar to [46]). This results in a local anomaly detection task with clusters of different densities and 10 local anomalies, which we refer to as *pen-local*.

**Letter Recognition** The UCI *letter* dataset contains originally 16 extracted features from the 26 letters of the English alphabet. This dataset has been preprocessed for unsupervised anomaly detection and was made publicly available [62]. Three letters have been chosen to form the normal class and anomalies have been sampled from the rest, which should result in a global anomaly detection task. The authors claim that the task is easy and they applied a procedure to make it more challenging: The number of dimensions was doubled to 32 by randomly concatenating normal instances of the three classes to all instances, including anomalies. This results in anomalies to have also some normal features making unsupervised anomaly detection more difficult. The resulting dataset has 1,600 instances including 6.25% anomalies.

**Speech Accent Data** The *speech* dataset was also provided by [62] and contains real world data from recorded English language. The normal class contains data from persons having an American accent whereas the outliers are represented from seven other speakers, having a different accent. The feature vector is the i-vector of the speech segment, which is a state-of-the-art feature in speaker recognition [65]. The dataset has 400 dimensions and is thus the task in our evaluation with the largest number of dimensions. It has 3,686 instances including 1.65% anomalies.

**Landsat Satellite** The *satellite* dataset comprises of features extracted from satellite observations. In particular, each image was taken under four different light wavelength, two in visible light (green and red) and two infrared images. The task of the original dataset is to classify the image into the soil category of the observed region. We defined the soil classes “red soil”, “gray soil”, “damp gray soil” and “very damp gray soil” as the normal class. From the semantically different classes “cotton crop” and “soil with vegetation stubble” anomalies are sampled. After merging the original training and test set into a single dataset, the resulting dataset contains 5,025 normal instances as well as 75 randomly sampled anomalies (1.49%) with 36 dimensions.

**Thyroid Disease** The *thyroid* dataset is another dataset from UCI machine learning repository in the medical domain. The raw patient measurements contain categorical attributes as well as missing values such that it was preprocessed in order to apply neural networks [66], also known as the “annthyroid” dataset. We make also use of this preprocessing, resulting in 21 dimensions. Normal instances (healthy non-hypothyroid patients) were taken from the training and test datasets. From the test set, we sampled 250 outliers from the two disease classes (subnormal function and hyperfunction) resulting in a new dataset containing 6,916 records with 3.61% anomalies.

**Statlog Shuttle** The *shuttle* dataset describes radiator positions in a NASA space shuttle with 9 attributes and was designed for supervised anomaly detection. Besides the normal “radiator flow” class, about 20% of the original data describe abnormal situations. To reduce the number of anomalies, we select the class 1 as normal and apply a stratified sampling for the classes 2, 3, 5, 6 and 7, similar to [67, 68]. Again, training and test set are combined in a single big dataset, which has as a result 46,464 instances with 1.89% anomalies.

**Object Images (ALOI)** The *aloi* dataset is derived from the “Amsterdam Library of Object Images” collection [63]. The original dataset contains about 110 images of 1000 small objects taken under different light conditions and viewing angles. From the original images, a 27 dimensional feature vector was extracted using HSB color histograms [38]. Some objects were chosen as anomalies and the data was down-sampled such that the resulting dataset contains 50,000 instances including 3.02% anomalies.

**KDD-Cup99 HTTP** As mentioned earlier, the *kdd99* dataset is often used for unsupervised anomaly detection. Similar to the shuttle dataset, this artificially created dataset was also designed for supervised anomaly detection. It basically contains simulated normal and attack traffic on an IP level in a computer network environment in order to test intrusion detection systems. In the past, the dataset was sometimes used by just sampling randomly from the attacks. Due to the nature of some attacks, for example DDoS, this represents not a point anomaly detection problem. To serve for our unsupervised evaluation purpose best, we decided to use HTTP traffic only (similar to [37]) and also limit DoS traffic from the dataset (similar to [69]). To this end, only 500 instances from these attacks are kept. This ensures that we do not have larger clusters among the anomalies. Furthermore, some of the features were adopted: First, “protocol” and “port” information were removed, since we select HTTP traffic only. The categorical “flags” feature was also removed and the remaining binary categorical features represented as 0 or 1 resulting in a total of 38 dimensions. The large-scale dataset contains finally 620,089 instances with only 0.17% anomalies. To our knowledge, this is the largest dataset in terms of instances used so far for unsupervised anomaly detection.

### Dataset Summary

All dataset characteristics are summarized in Table 1. With our dataset selection, we cover a broad spectrum of application domains including medical applications, intrusion detection, image and speech recognition as well as the analysis of complex systems. Additionally, the datasets cover a broad range of properties with regard to dataset size, outlier percentage and dimensionality. To our knowledge, this is the most comprehensive collection of unsupervised anomaly detection datasets for algorithm benchmarking. As already stated, we published the datasets to encourage researchers to compare their proposed algorithms with this work and hope to establish an evaluation standard in the community.

## Comparative Evaluation

Comparing the anomaly detection performance of unsupervised anomaly detection algorithms is not as straight forward as in the classical supervised classification case. In contrast to simply compare an accuracy value or precision/recall, the order of the anomalies should be taken into account. In classification, a wrongly classified instance is for sure a mistake. This is different in unsupervised anomaly detection. For example, if a large dataset contains ten anomalies and they are ranked among the top-15 outliers, this is still a good result, even if it is not perfect. To this end, a common evaluation strategy for unsupervised anomaly detection algorithms is to rank the results according to the anomaly score and then iteratively apply a threshold from the first to the last rank. This results in *N* tuple values (true positive rate and false positive rate), which form a single receiver operator characteristic (ROC). Then, the area under the curve (AUC), the integral of the ROC, can be used as a detection performance measure. A nice interpretation of the AUC is also given when following the proof from [70] and transform it into the anomaly detection domain: The AUC is then the probability that an anomaly detection algorithm will assign a randomly chosen normal instance a lower score than a randomly chosen anomalous instance. Hence, we think the AUC is a perfect evaluation method and ideal for comparison. However, the AUC only takes the ranking into account and completely neglects the relative difference of the scores among each other. Other measures can be used to cope with this shortcoming by using more sophisticated rank comparisons. Schubert et al. [38] compares different rank correlation methodologies, for example Spearman’s ρ and Kendall’s τ as an alternative to AUC with a focus on targeting outlier ensembles. A second possible drawback of using AUC might be that it is not ideal for unbalanced class problems and methods like area under precision-recall curve or Matthews correlation coefficient could possibly better emphasize small detection performance changes. Nevertheless, AUC based evaluation has been evolved to be the de facto standard in unsupervised anomaly detection, most likely due to its practical interpretability, and thus also serves as the measure of choice in our evaluation.

**Fig 8:**
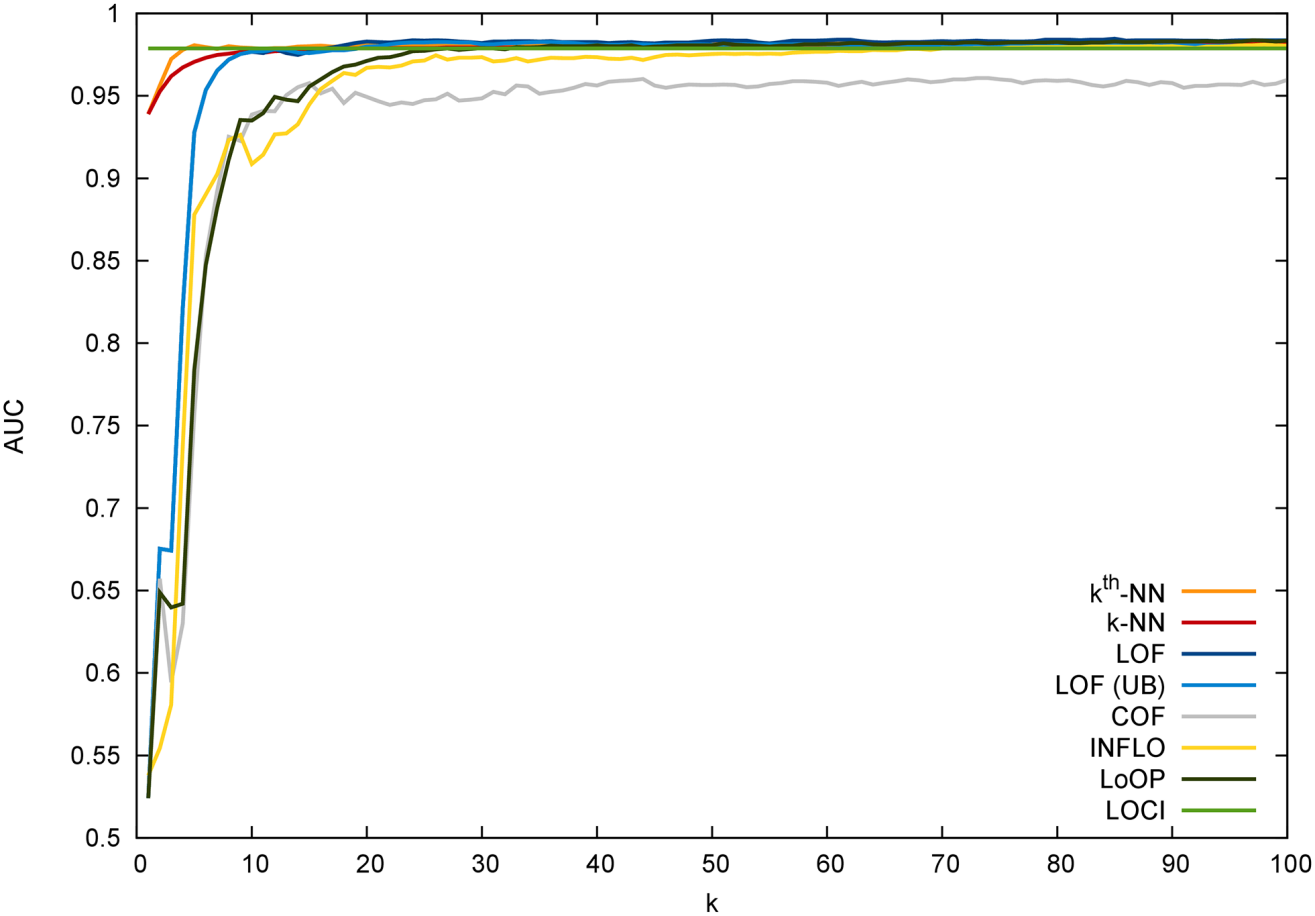
The AUC values for the nearest-neighbor based algorithms on the breast-cancer dataset. It can be seen that *k* values smaller than 10 tend to result in poor estimates, especially when considering local anomaly detection algorithms. Please note that the AUC axis is cut off at 0.5.

Please note that the AUC, when it is used in a traditional classification task, typically involves a parameter, for example *k*, to be altered. In unsupervised anomaly detection, the AUC is computed by varying an outlier threshold in the ordered result list. As a consequence, if a parameter has to be evaluated (for example different *k*), this yields to multiple AUC values. Another important question in this context is how to evaluate *k*, the critical parameter for most of the nearest-neighbor and clustering-based algorithms. In most publications, researchers often fix *k* to a predefined setting or choose “a good *k*” depending on a favorite outcome. We believe that the latter is not a fair evaluation, because it somehow involves using the test data (the labels) for training. In our evaluation, we decided to evaluate many different *k*’s between 10 and 50 and finally report the averaged AUC as well as the standard deviation. In particular, for every *k*, the AUC is computed first by varying a threshold among the anomaly scores as described above. Then, the mean and standard deviation for all these AUCs is reported. This procedure basically corresponds to a random-*k*-picking strategy within the given interval, which is often used in practice when *k* is chosen arbitrarily. The lower bound of 10 was chosen because of statistical fluctuations occurring below. For the upper bound, we set a value of 50 such that it is still suitable for our smaller datasets. We are aware, that one could argue to increase the upper bound for the larger datasets or even make it smaller for the small datasets like breast-cancer. Fig 8 shows a broader evaluation of the parameter *k* illustrating that our lower and upper bound is in fact useful.

### Results of the Nearest-neighbor based Algorithms

Table 2 shows the results of the nearest-neighbor based algorithms. The AUC values are averaged for 10 ≤ *k* ≤ 50 and the standard deviation is shown. For LOCI, which does not rely on *k*, only one result is available. As already stated, LOCI is very computationally intensive and could not be computed within a reasonable time for larger datasets. Since aLOCI is not a deterministic algorithm, it was run 20 times and the average result was taken. For both, LOCI and aLOCI, the recommended parameter settings from the authors were used [47]. When comparing aLOCI with the other algorithms, the results are significantly worse: It is often the worst performing algorithm and the high standard deviation of the results additionally shows less stability of the results. Thus, the use of LOCI and aLOCI is at least questionable in practice. In this context, recall that it is not possible on unlabeled data to determine whether a non-deterministic aLOCI outcome is good or not for a practical application. Furthermore, it can be observed from the table that the results of the two *k*-NN and LOF variants do not differ much and there is no clear winner or recommendation. At least for LOF, this result was not expected, since the authors claimed that the LOF-UB ensemble performs better in general [43].

**Table 2:** The results of the nearest-neighbor based algorithms showing the AUC and the standard deviation for 10 ≤ *k* ≤ 50 for all of the 10 datasets. Due to the computational complexity, LOCI could not be computed for larger datasets.

Alg.	b-cancer	pen-global	pen-local	letter	speech	satellite	thyroid	shuttle	aloi	kdd99
*k*-NN	0.9791±0.0010	**0.9872**±0.0055	0.9837±0.0018	0.8719±0.0176	0.4966±0.0101	**0.9701**±0.0007	0.5956±0.0125	0.9424±0.0069	0.6502±0.0191	0.9747±0.0045
*k^th^*-NN	0.9807±0.0008	0.9778±0.0142	0.9757±0.0069	0.8268±0.0228	0.4784±0.0007	0.9681±0.0015	0.5748±0.0128	0.9434±0.0101	0.6177±0.0189	**0.9796**±0.0035
LOF	**0.9816**±0.0024	0.8495±0.0679	**0.9877**±0.0016	0.8673±0.0271	0.5038±0.0215	0.8147±0.1126	0.6470±0.0192	0.5127±0.0129	0.7563±0.0135	0.5964±0.0284
LOF-UB	0.9805±0.0020	0.8541±0.0777	0.9876±0.0013	0.9019±0.0030	0.5233±0.0134	0.8425±0.0839	0.6663±0.0103	0.5182±0.0124	0.7713±0.0045	0.5774±0.0159
COF	0.9518±0.0054	0.8695±0.1261	0.9513±0.0134	0.8336±0.0228	0.5218±0.0287	0.7491±0.0952	0.6505±0.0154	0.5257±0.0086	0.7857±0.0118	0.5548±0.0236
INFLO	0.9642±0.0171	0.7887±0.0540	0.9817±0.0024	0.8632±0.0250	0.5017±0.0191	0.8272±0.0761	0.6542±0.0158	0.4930±0.0175	0.7684±0.0142	0.5524±0.0222
LoOP	0.9725±0.0123	0.7684±0.0994	0.9851±0.0068	**0.9068**±0.0078	**0.5347**±0.0343	0.7681±0.0433	**0.6893**±0.0149	0.5049±0.0035	**0.7899**±0.0093	0.5749±0.0275
LOCI	0.9787	0.8877	—	0.7880	0.4979	—	—	—	—	—
aLOCI	0.8105±0.0883	0.6889±0.0345	0.8011±0.0615	0.6208±0.0220	0.4992±0.0348	0.8324±0.0372	0.6174±0.0221	**0.9474**±0.0379	0.5855±0.0143	0.6552±0.0458

**Fig 9:**
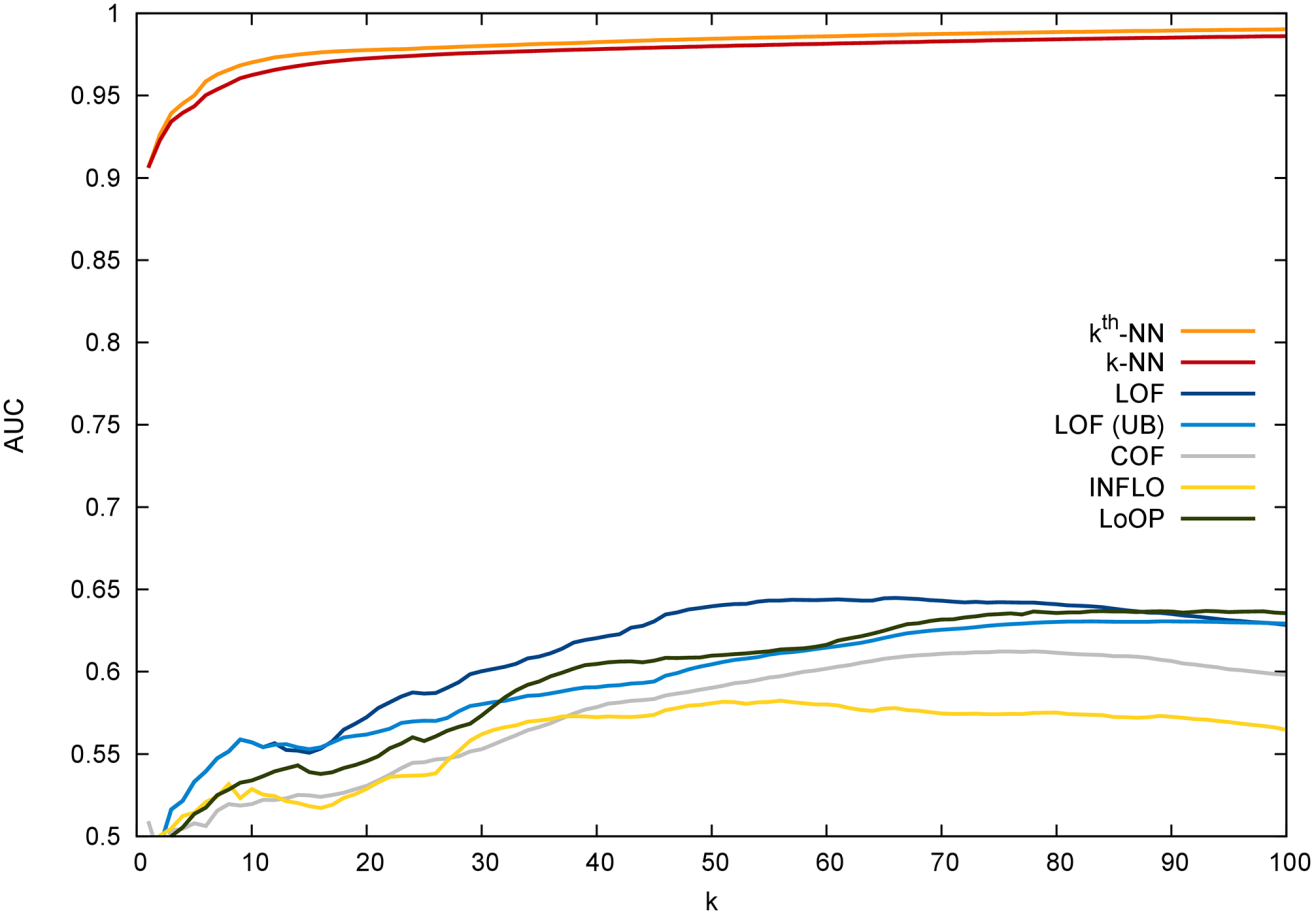
The AUC values for the large kdd99 dataset for 0 < *k* < 100. It can be easily seen that the performance of local anomaly detection algorithms is poor for this global anomaly detection challenge.

Another very important finding from our evaluation can be inferred when comparing the two columns of the pen-global/local datasets. It can be seen that the local anomaly detection algorithms perform much worse on the global anomaly detection task. Also, the same observation could be made on the (global) shuttle and kdd99 dataset. For the latter, Fig 9 illustrates the superiority of *k*-NN compared to the local algorithms. A final observation is the general poor performance of all algorithms on the high-dimensional speech dataset. An AUC of 0.5 shows that the detection performance is as good as a random guess. When we looked into the results in more detail, we could observe that the performance for very small *k* values is much better (for almost all algorithms). The *k* values of 2, 3 and 4 show AUCs of up to 0.78 with a quick drop when *k* is larger. We suspect that due to the high number of dimensions, the curse of dimensionality leads to poor results for *k* > 5.

### Results of the Clustering-based Algorithms

Table 3 summarizes the results for the clustering-based anomaly detection algorithms. For every algorithm, we used the parameter settings as recommended by the authors as a default: The parameters separating into small/large clusters for CBLOF and uCBLOF are *α* = 95, *β* = 5, and for LDCOF and CMGOS *γ* = 0.3. Additionally, for CMGOS the estimated amount of normal instances is *p_n_* = 0.975. In order to make the clustering-based algorithm evaluation as comparable as possible, we used the same clustering result for every algorithm. For example, for *k* = 10 we applied *k*-means and stored the resulting centroids and cluster belongings. Then, all algorithms use this result as a basis for computing their scores. Since *k*-means is also a non-deterministic algorithm, we ran it 10 times on the same data and follow a common strategy by picking the most stable clustering result.

**Table 3:** The results of the clustering-based algorithms showing the AUC and the standard deviation for different initial *k* (10 ≤ *k* ≤ 50). The last row shows a comparison with the best nearest-neighbor method for the dataset.

Alg.	b-cancer	pen-global	pen-local	letter	speech	satellite	thyroid	shuttle	aloi	kdd99
CBLOF	0.2983±0.1492	0.3190±0.1155	0.6995±0.1407	0.6792±0.0386	0.5021±0.0680	0.5539±0.0692	0.5825±0.0384	0.9037±0.1263	0.5393±0.0154	0.6589±0.2098
uCBLOF	**0.9496**±0.0390	**0.8721**±0.0511	0.9555±0.0109	0.8192±0.0231	0.4692±0.0029	**0.9627**±0.0038	0.5469±0.0212	**0.9716**±0.0324	0.5575±0.0061	**0.9964**±0.0016
LDCOF	0.7645±0.1653	0.5948±0.0825	0.9593±0.0145	0.8107±0.0244	0.4366±0.0099	0.9522±0.0325	0.5703±0.0232	0.8076±0.1814	0.5726±0.0146	0.9873±0.0034
CMGOS-Red	0.9140±0.0815	0.5693±0.1000	**0.9727**±0.0141	0.7711±0.0614	0.5077±0.0158	0.9054±0.0233	0.4395±0.0402	0.5425±0.2446	0.5852±0.0161	0.7265±0.1027
CMGOS-Reg	0.8992±0.0643	0.6994±0.0681	0.9449±0.0510	**0.8902**±0.0200	**0.5081**±0.0161	0.9056±0.0233	0.6587±0.0268	0.5679±0.2402	**0.5855**±0.0161	0.9797±0.0080
CMGOS-MCD	0.9196±0.0830	0.6265±0.0969	0.9038±0.0511	0.7848±0.0485	—	0.9120±0.0520	**0.8014**±0.0436	0.6903±0.1670	0.5547±0.0160	0.9696±0.0416
Best NN	**0.9816**±0.0024	**0.9872**±0.0055	**0.9877**±0.0016	**0.9068**±0.0078	**0.5347**±0.0343	**0.9701**±0.0007	0.6893±0.0149	0.9474±0.0379	**0.7899**±0.0093	0.9796±0.0035

The results show that CBLOF performs poorly in most cases. Especially on the smaller datasets, the algorithm is even worse than random guessing. Since this behavior is very suspicious, we looked into the results in detail: AUCs from 0.10 to 0.94 occurred, but no correlation to *k* could be found. Our suspicion concerning the results is again the possible flaw of weighting the scores by number of members in the cluster—especially on small datasets the influence seems disadvantageous. Simply removing the weighting yields to much better results, proven by the results of uCBLOF. Similar to our observation on the nearest-neighbor based algorithms, again local methods tend to perform worse on global anomaly detection tasks. Concerning the different robust estimations of the covariance matrix for CMGOS, two techniques seem to perform well: GMGOS-Red as well as CMGOS-MCD. Due to the much higher computational complexity, we recommend to use CMGOS-Red. The high number of dimensions in the speech dataset causes CMGOS-MCD to not complete within a reasonable amount of time.

Special attention should be payed on the last row of the table, where the best nearest-neighbor method is listed for comparison. It can be seen that nearest-neighbor based unsupervised anomaly detection performs better in general. However, a much more severe issue is from our point of view the reliability indicated by the corresponding standard deviations. In practice, when the parameter *k* cannot be determined and needs to be fixed to a certain value, nearest-neighbor based algorithms generate much more stable results.

### Results of other Algorithms

In the remaining, the four algorithms are evaluated which do not belonging to one of the groups above. Table 4 shows the results for the statistical HBOS, the subspace rPCA algorithm as well as the results for the two one-class SVM methods. The evaluation of HBOS is performed similar to the previous algorithms, whereas here *k* refers to the number of bins used. The constraint 10 ≤ *k* ≤ 50 was also used for HBOS. rPCA has no critical parameter *k* to be evaluated, but as described earlier, a different amount of components can be used. In total, we applied four different strategies: (1) Using all components, (2) Using major components only, (3) Using minor components only and (4) Using major and minor while neglecting the ones in the middle. We found that the results of the four strategies are very similar (for some datasets even identical) and therefore reported the averaged AUC in the table. The parameters *ν* for the one-class SVM as well as *β* for the enhanced *η* one-class SVM were also altered in the range 0.2 ≤ *ν* ≤ 0.8 and the average AUC was reported. Choosing these parameters seems less critical than choosing a correct *k* for other algorithms—it seems that a setting of *β*/*ν* = 0.5 is a good choice on average. However, the parameter value should not be set too small to avoid an incorrect density estimation. Furthermore, a Gaussian kernel with automatic gamma tuning was used. Tuning this parameter automatically is ideal for unsupervised anomaly detection, but requires an significant amount of computation time.

**Table 4:** The AUC results of the remaining unsupervised anomaly detection algorithms. Four different strategies for keeping the components have been used for rPCA, while for HBOS the number of different bins was altered.

Alg.	b-cancer	pen-global	pen-local	letter	speech	satellite	thyroid	shuttle	aloi	kdd99
HBOS	**0.9827**±0.0016	0.7477±0.0206	0.6798±0.0249	0.6216±0.0073	0.4708±0.0030	0.9135±0.0047	**0.9150**±0.0123	**0.9925**±0.0039	0.4757±0.0010	**0.9990**±0.0007
rPCA	0.9664±0.0000	0.9375±0.0001	0.7841±0.0151	0.8095±0.0029	0.5024±0.0000	0.9461±0.0023	0.6574±0.0036	0.9963±0.0000	0.5621±0.0000	0.7371±0.0000
oc-SVM	0.9721±0.0102	0.9512±0.0436	0.9543±0.0130	0.5195±0.0382	0.4650±0.0021	0.9549±0.0021	0.5316±0.0152	0.9862±0.0002	0.5319±0.0021	0.9518±0.0050
*η*-oc-SVM	0.9581±0.0311	0.8993±0.0387	0.9236±0.0140	0.7298±0.1365	0.4649±0.0026	0.9430±0.0058	0.5625±0.0088	0.9848±0.0019	0.5221±0.0025	0.7945±0.0000
Best NN	0.9816±0.0024	**0.9872**±0.0055	**0.9877**±0.0016	**0.9068**±0.0078	**0.5347**±0.0343	**0.9701**±0.0007	0.6893±0.0149	0.9474±0.0379	**0.7899**±0.0093	0.9796±0.0035
Best Cluster	0.9496±0.0390	0.8721±0.0511	0.9727±0.0141	0.8902±0.0200	0.5081±0.0161	0.9627±0.0038	0.7843±0.0437	0.9716±0.0324	0.5855±0.0161	0.9964±0.0016
Best Alg.	HBOS	*k*-NN	LOF	LoOP	LoOP	*k*-NN	HBOS	HBOS	COF	HBOS

The results of the four algorithms are very diverse. For us, the biggest surprise was the good performance of HBOS on the larger (global) datasets. For kdd99, the result is almost perfect while on the thyroid dataset, it outperformed all the other algorithms by far. The other algorithm with a rather simple (linear) model, rPCA, performed average. One exception is the shuttle dataset, where rPCA obtains together with HBOS the best results. One-class SVMs turned out to be not outstanding algorithms and results are average. When comparing the enhanced *η* one-class SVM with the regular one, the latter seems to perform better.

### Computation Time Comparison of Algorithms

If determinable, the theoretical computational complexity of the evaluated algorithms was already discussed. However, in practice, the actual computation times may still be quite different from each other. For this reason, the computation times were measured and are listed in Table 5. Please note that the listed times are measured in seconds for the first nine datasets and in minutes for the last column, the large kdd99 dataset. The time was measured on a single thread basis. For the clustering-based algorithms, the computation time depends strongly on the number of clusters. For that reason, the times for 10 and 50 clusters are listed separately for each algorithm.

**Table 5:** Comparing the computation time of the different algorithm show huge differences, especially for the larger datasets. The unit of the table is seconds for the first nine columns and minutes for the last dataset (kdd99).

Alg.	b-cancer	pen-global	pen-local	letter	speech	satellite	thyroid	shuttle	aloi	kdd99
*k*-NN	<0.1	<0.1	2.4	0.3	5.7	2.0	2.6	106	166	538
*k^th^*-NN	<0.1	<0.1	2.4	0.3	5.8	2.0	2.6	105	165	538
LOF	<0.1	<0.1	2.4	0.3	5.8	2.0	2.7	105	165	538
LOF-UB	<0.1	<0.1	2.6	0.3	5.9	2.1	2.8	107	167	539
COF	<0.1	0.1	2.8	0.5	9.0	2.5	3.1	107	169	539
INFLO	<0.1	<0.1	2.4	0.3	5.8	2.0	2.6	105	165	538
LoOP	<0.1	<0.1	2.5	0.3	5.8	2.0	2.6	105	165	538
LOCI	18	240	—	2572	25740	—	—	—	—	—
aLOCI	0.5	1.8	90	12.7	9.5	56	30	73	1137	298
CBLOF/LDCOF 10	<0.1	0.1	1.5	0.6	24.8	4.0	1.0	6.9	39.1	5.01
CBLOF/LDCOF 50	0.1	0.2	3.7	5.9	24.7	5.2	4.4	10.3	74.6	16.14
CMGOS-Red 10	0.5	0.2	1.7	1.1	82	4.6	1.2	7.0	40	5.15
CMGOS-Red 50	0.1	0.5	4.3	1.7	49	8.2	4.6	10.6	77	16.25
CMGOS-Reg 10	0.4	0.2	1.7	1.1	83	4.6	1.3	7.0	40	5.19
CMGOS-Reg 50	0.1	0.5	4.3	1.7	49	8.1	5.4	10.6	77	16.29
CMGOS-MCD 10	159	211	863	759	—	3821	1967	354	3003	491
CMGOS-MCD 50	735	519	1045	1441	—	4041	4159	1525	10933	8745
HBOS	<0.1	<0.1	<0.1	<0.1	0.5	<0.1	<0.1	<0.1	0.4	0.06
rPCA	<0.1	<0.1	0.2	<0.1	9.2	0.1	<0.1	0.3	1.5	21.8
oc-SVM	0.3	0.5	31	8.5	807	28	26	19639	59531	5480
*η*-oc-SVM	0.3	0.4	70	8.2	745	24	27	19087	58559	3310

Except for the very demanding LOCI algorithm, it can be seen that the computation for the small datasets is sufficiently fast, such that the choice of an appropriate algorithm should focus on detection performance, not on runtime. On the contrary, for large datasets, computation time differences are significant. For example, for the largest dataset kdd99, the fastest algorithm HBOS took less than 4 seconds, whereas the slowest GMGOS-MCD took more than 6 days.

In general, it can be observed that nearest-neighbor based algorithms have almost identical runtimes. This is due to the fact, that the nearest-neighbor search is responsible for most of the computation time, whereas the (different) computation of the scores itself has almost no influence. Furthermore, it can be confirmed that the clustering-based algorithms (except for CMGOS-MCD) are faster than the nearest-neighbor based algorithms with the quadratic search complexity. Please keep in mind that the time includes the execution of ten different runs of the underlying *k*-means algorithm. At this point, we would like to state again, that the use of CMGOS-MCD is not recommended. HBOS is by far the fastest algorithm among all, which is due to its very simple idea of assuming independence of the features. The comparable high runtimes of the SVM based algorithms are mainly based on the automatic gamma tuning, which has a quadratic complexity. For example, for the aloi dataset, the gamma tuning takes about 16 hours, whereas the core SVM training is only 30 seconds for the *η* one-class SVM and 16 minutes for the regular one-class SVM.

## Conclusion

A comprehensive evaluation of 19 different unsupervised anomaly detection algorithms on 10 datasets from different application domains has been performed for the first time. The algorithms have been released as an open source extension for the RapidMiner data mining software (available at http://git.io/vnD6n). Additionally, the datasets have been made publicly available (http://dx.doi.org/10.7910/DVN/OPQMVF) and therefore a foundation for a fair and standardized comparison for the community was introduced. Besides supporting the unsupervised anomaly detection research community, we also believe that our study and our implementation is useful for researchers from neighboring fields. Now, it is easy to apply the discussed methods on new data. The broad variety of our evaluation datasets might guide for appropriate algorithm selection in new application domains.

In particular, our findings include that local anomaly detection algorithms, such as LOF, COF, INFLO and LoOP tend to perform poorly on datasets containing global anomalies by generating many false positives. The usage of these algorithms should be avoided if it is known that the task is to detect global anomalies only. On the contrary, global anomaly detection algorithms perform at least average on local problems. This yields in our recommendation to select a global anomaly detection algorithm if there is no further knowledge about the nature of anomalies in the dataset to be analyzed.

As a general detection performance result, we can conclude that nearest-neighbor based algorithms perform better in most cases when compared to clustering algorithms. Also, the stability concerning a not-perfect choice of *k* is much higher for the nearest-neighbor based methods. The reason for the higher variance in clustering-based algorithms is very likely due to the non-deterministic nature of the underlying *k*-means clustering algorithm. Despite of this disadvantage, clustering-based algorithms have a lower computation time. As a conclusion, we recommend to prefer nearest-neighbor based algorithms if computation time is not an issue. If a faster computation is required for large datasets, for example in a near real-time setting, clustering-based anomaly detection might be the method of choice. For small datasets, clustering-based methods should be avoided.

Among the nearest-neighbor based methods, the global *k*-NN algorithm is a good candidate on average. Although LoOP was the best performing nearest-neighbor based algorithm on four datasets, it unfortunately fails significantly one some datasets. Especially for the global anomaly detection problems this algorithm should be totally avoided. Besides our recommendation for *k*-NN, LOF is also a good candidate if it is previously known that the anomaly detection problem to be solved involves local anomalies.

Concerning the clustering-based algorithms, the simple uCBLOF algorithm also shows on average good performance for all datasets, illustrating that a more sophisticated and compute intense density estimation is not necessarily required. In terms of computational complexity, clustering-based algorithms are faster than their nearest-neighbor competitors. However, in practice, we advice to restart the underlying *k*-means algorithm multiple times in order to obtain a stable clustering outcome. This procedure unfortunately often takes away the advantage of the theoretical speedup, which leads on the small datasets even to longer runtimes compared with the nearest-neighbor based algorithms. Nevertheless, when processing speed is very important or a clustering model can be updated in a data streaming application, a clustering-based algorithm might be used. Besides our recommendation for uCBLOF, CMGOS-Reg also seems to perform reliable on most of the datasets. On the contrary, the original CBLOF algorithm should be avoided due to an algorithm design flaw. Also, the CMGOS with the subspace-based MCD density estimation should not be the first choice, since the density estimation is too slow and detection performance is worse.

The statistical algorithm HBOS, which assumes independence of the features, surprisingly showed very good results in our evaluation. It is even the best performing algorithm on four out of our 10 datasets. Due to the very fast computation time, especially for large datasets, we highly recommend to give it a try on large-scale datasets when looking for global anomalies.

One dataset with 400 dimensions was a big challenge for all of the algorithms, most likely due to the curse of dimensionality. In this context, only nearest-neighbor based algorithms with a very small *k* < 5 were useful at all. Since in unsupervised anomaly detection *k* can typically not be determined, we might conclude that unsupervised anomaly detection fails on such a high number of dimensions.

As a general summary for algorithm selection, we recommend to use nearest-neighbor based methods, in particular *k*-NN for global tasks and LOF for local tasks instead of clustering-based methods. If computation time is essential, HBOS is a good candidate, especially for larger datasets. A special attention should be paid to the nature of the dataset when applying local algorithms, and if local anomalies are of interest at all in this case. We have summarized our recommendations for algorithm selection in Table 6 with respect to the anomaly detection performance (accuracy), the stability of the scoring (deterministic), the sensitivity to parameters, the computation time for larger datasets (speed) and whether the algorithm is applicable for datasets having global anomalies only. Please note, that the judgments in the table assume that the general recommendations as given above are followed.

**Table 6:** Recommendations for algorithm selection. Qualitatively judgments are given from very bad (− −) over average (o) to very good (++).

Alg.	accuracy	deterministic	sensitivity	speed	global detection
*k*-NN	++	++	+	o	++
LOF	++	++	+	o	− −
COF	−	++	+	o	− −
INFLO	o	++	+	o	− −
LoOP	++	++	+	o	− −
LOCI	o	++	++	− −	− −
aLOCI	−	− −	− −	o	−
CBLOF	− −	o	o	+	− −
uCBLOF	++	o	o	+	++
LDCOF	−	o	o	+	o
CMGOS-Red	o	o	o	+	−
CMGOS-Reg	o	o	o	+	+
CMGOS-MCD	−	−	−	− −	o
HBOS	+	++	o	++	++
rPCA	o	++	+	+	o
oc-SVM	o	+	+	− −	+
*η*-oc-SVM	o	+	+	− −	o
